# Superior Effects of Antiretroviral Treatment among Men Who have Sex with Men Compared to Other HIV At-Risk Populations in a Large Cohort Study in Hunan, China

**DOI:** 10.3390/ijerph13030283

**Published:** 2016-03-04

**Authors:** Shu Su, Xi Chen, Limin Mao, Jianmei He, Xiuqing Wei, Jun Jing, Lei Zhang

**Affiliations:** 1 Melbourne Sexual Health Centre, Alfred Health, Melbourne, VIC 3004, Australia; ssu27@student.monash.edu; 2 School of Public Health and Preventive Medicine, Faculty of Medicine, Nursing and Health Sciences, Monash University, Melbourne, VIC 3004, Australia; 3 Hunan Provincial Center for Disease Control and Prevention, Changsha 410005, Hunan, China; chenxi161@sohu.com (X.C.); jmhe69@126.com (J.H.); isqq2011@163.com (X.W.); 4 Center for Social Research in Health, Faculty of Arts and Social Science at the University of New South Wales, Sydney, NSW 2052, Australia; limin.mao@unsw.edu.au; 5 Comprehensive AIDS Research Center, Tsinghua University, Beijing 100084, China; jingjun@tsinghua.edu.cn; 6 Central Clinical School, Faculty of Medicine, Nursing and Health Sciences, Monash University, Melbourne, VIC 3004, Australia

**Keywords:** CD4, viral load, treatment failure, MSM, HIV, China

## Abstract

This study assesses association between CD4 level at initiation of antiretroviral treatment (ART) on subsequent treatment outcomes and mortality among people infected with HIV via various routes in Hunan province, China. Over a period of 10 years, a total of 7333 HIV-positive patients, including 553 (7.5%) MSM, 5484 (74.8%) heterosexuals, 1164 (15.9%) injection drug users (IDU) and 132 (1.8%) former plasma donors (FPD), were recruited. MSM substantially demonstrated higher initial CD4 cell level (242, IQR 167–298) than other populations (Heterosexuals: 144 IQR 40–242, IDU: 134 IQR 38–224, FPD: 86 IQR 36–181). During subsequent long-term follow up, the median CD4 level in all participants increased significantly from 151 cells/mm^3^ (IQR 43–246) to 265 cells/mm^3^ (IQR 162–380), whereas CD4 level in MSM remained at a high level between 242 and 361 cells/mm^3^. Consistently, both cumulative immunological and virological failure rates (10.4% and 26.4% in 48 months, respectively) were the lowest in MSM compared with other population groups. Survival analysis indicated that initial CD4 counts ≤200 cells/mm^3^ (AHR = 3.14; CI, 2.43–4.06) significantly contributed to HIV-related mortality during treatment. Timely diagnosis and treatment of HIV patients are vital for improving CD4 level and health outcomes.

## 1. Introduction

Antiretroviral therapy (ART) can significantly reduce mortality and prevent opportunistic infections in HIV-positive patients by strengthening immune response that suppress virus replication [1,2]. Timely diagnosis and treatment initiation have been recognized as an effective approach to prevent HIV transmission from people living with HIV (PLHIV) to the susceptible population [3,4]. In 2014, UNAIDS declared its ambitious 90-90-90 goals to enable 90% of people living with HIV to be diagnosed, 90% of diagnosed individuals to receive ART and 90% of those on ART have successful viral suppression by 2020 [4]. ART plays a vital role in achieving these goals and ending the HIV epidemics.

China launched its national ART program in 2002 with a treatment threshold of CD4 cell level below 200 cells/mm^3^. This threshold has increased gradually over time in national treatment guidelines [5]. In 2004, China implemented its “Four Frees and One Care” policy, which commited to the provision of free ART, voluntary counselling and testing, prevention of mother-to-child transmission, education for AIDS orphans and economic assistance for PLHIV [6]. The ART program has since rapidly expanded and the treatment threshold was raised to 350 cells/mm^3^ in 2008 [7]. Previous studies have reported that high CD4 count at ART initiation reduces the risk of treatment failure and death [8,9,10]. Not until recently, with dramatic scale-up of government input into the program, were PLHIV recommended to initiate treatment early, irrespective of their CD4 cells level. This was reflected by a substantial surge in the number of people receiving treatment [6,11].

HIV prevalence among men who have sex with men (MSM) in China has reportedly increased from 1.8% in 2000 to 7.3% in 2013 [12,13]. MSM are a known high-risk population for HIV transmission due to frequent unprotected anal intercourse and multiple concurrent sexual partnerships [13,14]. Diagnosis rate in MSM remains low in China, as estimated 87% HIV-infected cases among MSM remain unidentified [15]. HIV prevalence among MSM has exceeded other risk populations, such as injection drug users (IDU) and female sex workers, in most parts of China [16]. Treatment failure rate and mortality are also reportedly higher among MSM compared with other at-risk populations [17]. The situation can be attributed to delayed treatment that causes severely impaired immune response to infection [18,19]. Other studies also reported higher drug resistance level in MSM [20,21,22]. Hence, exploring whether MSM with higher CD4 cell counts at ART initiation can improve treatment outcomes compared with other at-risk populations may provide valuable insights into the impacts of ART on MSM living with HIV.

We conducted our study in the Hunan province, Central China, which has a population of 66 million [23]. The HIV epidemic in Hunan largely resembles the epidemic nationwide. However, Bill and Melinda Gates Foundation has funded a large-scale intervention program to provide free rapid HIV screening for MSM during 2008–2011, aiming to increase testing rate among MSM to above 60% (Unpublished). As a result, Hunan is the only Chinese province that reported a three-fold increase in HIV testing coverage among MSM (from 16.6% to 58.6%) during the intervention period [24]. This implies a higher diagnosis rate and likely early treatment initiation in MSM in Hunan. This setting also provides a unique opportunity to compare the differences in treatment outcomes between MSM and 7000 other PLHIV who were infected via other routes in the ART program. This research aims to investigate the impacts of higher CD4 counts at ART initiation on MSM by comparing mortality and treatment failure rates with those of heterosexuals, IDU and former plasma donor (FPD).

## 2. Materials and Methods

### 2.1. Study Design and Data Collection

China established its national ART database in 2002, which was then managed by the National Centre for AIDS/STD Control and Prevention (NCAIDS), Chinese Centre for Disease Control and Prevention (CDC) [25]. The database consisted of five standard aspects: initial patient assessment, treatment follow-up, treatment regimen information, treatment follow-up and transfer of care status. Local CDC staff and grass root healthcare providers were trained to collect and upload required information of patients to the national database through DataFax, which is a client-server data management system for storage, extraction and management of HIV patient data. Each patient’s information was reviewed twice by the national database managers to check for missing data and logical errors, with queries returned to the local CDC for correction.

In this study, data was collected from Hunan province of national free ART database. All PLHIV older than 18 who newly enrolled in ART from any treatment provision sites in Hunan province were included in this study. The cohort started from 1 January 2003 and ended at the 31 December 2013. We only used the baseline, follow-up and treatment status information for the purpose of this study.

### 2.2. Clinical Laboratory Testing at Baseline

Demographic information, mode of infection, medical history and laboratory test results of patients were collected only once by the health workers at ART initiation, but CD4 count level was monitored after positive confirmation and throughout the course of treatment. Viral load was only recorded at baseline and followed up on a voluntary basis as the detection cost was high. Patients who have experienced immunological failure previously were recommended to receive viral load test, but recently more patients with strong health consciousness also requested for viral load tests. Disease progression stages were classified according to WHO guidelines [26]. Days from diagnosis to treatments was calculated as the difference between the date of diagnosis and ART initiation date.

### 2.3. Disease Progression Indicators

Three key outcome indicators were collected to evaluate the effects of ART:

(1) CD4 and HIV viral levels. The median with interquartile range (IQR) of CD4 counts and viral load were calculated every three months in the first year then every six months over the remaining course of follow-up.
(2) Treatment failure (including both immunological and virological failure). Immunological failure was defined as CD4 Cell count of the patient fell below 100 cells/mm^3^ after receiving ART for three months without a concomitant diseases that may sharply decrease the CD4 counts [27]; virological failure was defined as plasma viral load above 1000 copies/mL based on two consecutive viral load measurements after 3 months of treatment [28].

Considering that the majority of participants (5411, 73.8%) initiated ART after 2010, only a few people have CD4 and viral load test more than 43 months of follow-up, we categorized records beyond 43 months as a single time interval.

(3) HIV-related mortality. All death cases were recorded until the end of the study (December 2013). Mortality was compared across different CD4 count strata and transmission route categories. Factors associated with mortality were identified with Cox regression.

### 2.4. Statistical Analysis

All surveyed patients were stratified according to their transmission route (FPD, MSM, Heterosexual and IDU). All data were entered and analysed using statistical software SAS 9.2 (Statistical Analysis System). Descriptive and inferential statistical analyses were performed. The mean, median and IQR were used to summarise numerical variables; whereas frequencies and percentages were used to describe categorical variables. A linear regression model was constructed to verify whether the ascending trend of CD4 Cell counts during the follow-up was significant. The survival analysis was performed to compare the survival probability across transmission groups. A multivariable Cox proportional hazards model was used to identify the hazard factor contributing to HIV-related mortality, immunological failure and virological failure. A *p*-value of less than 0.05 was considered significant in the final model. The patients with missing data were included in the “unknown group”.

### 2.5. Ethical Considerations

This study was reviewed and approved by the Monash University Human Research Ethics Committee (approval number CF15/4321–2015001862). No personal information about the patients were disclosed in this research. The collected data were analysed only for the purposes of this study. Besides the members of the experimental group, no other individual, group, or institution had access to this data. No further informed consent about this study was required.

## 3. Result

### 3.1. Demographic Characteristics and CD4 Level at Baseline

**Figure 1:**
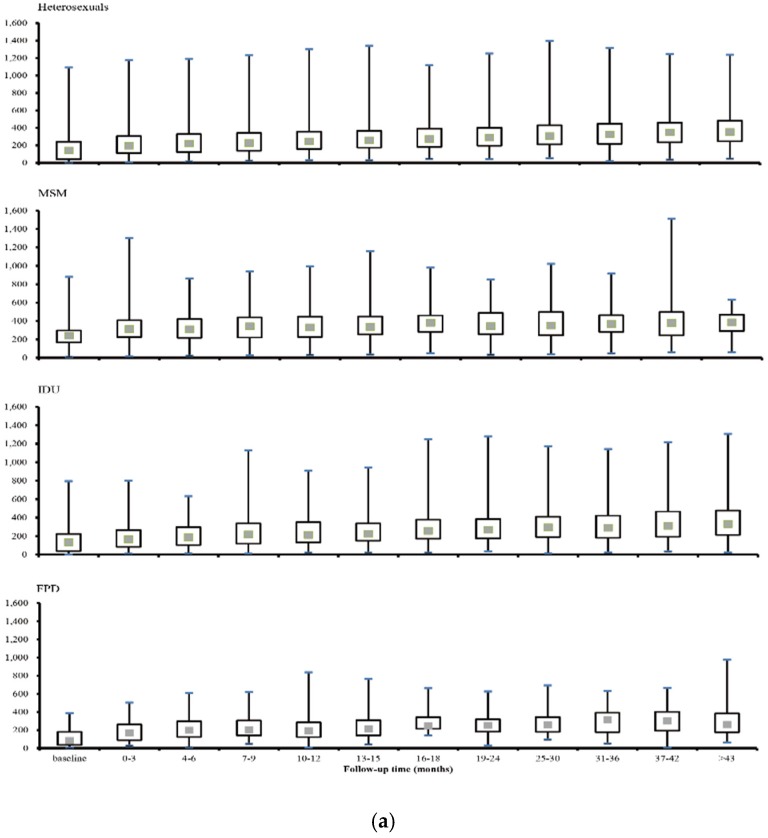

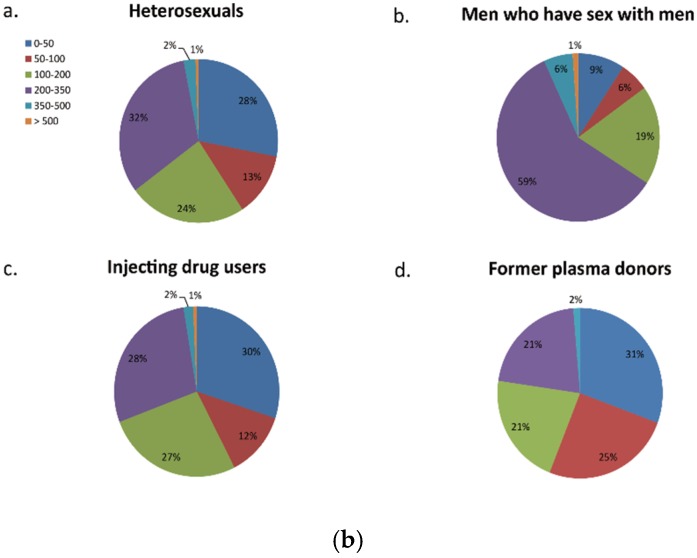
(**a**) CD4 T cell variation in populations stratified by transmission routes since initiation of antiretroviral treatment (ART); (The grey dot represents the median value, whereas the rectangular box represents the interquartile range of the distribution. The maximum and minimum values are marked with blue bars in each box plot); (**b**) Composition of CD4 counts in populations stratified by transmission routes, *i.e.,* a. heterosexuals; b. men who have sex with men; c. injecting drug users; d. former plasma donors, at ART initiation.

A total of 7333 PLHIV (5484 heterosexuals, 553 MSM, 1164 IDU and 132 FPD) were included in this study over the period of 2003–2013. A maximum of 82 months follow-up has been recorded after ART enrolment (median 27 months; IQR, 12–39 months). At treatment initiation, the sample has a median age of 40 years (IQR, 32–49). The median CD4 cell count was 151 cells/mm^3^ (IQR, 43–246). Female has significantly higher CD4 count than male (Median CD4 164 cells/mm^3^ *vs.* 138 cells/mm^3^, respectively, *p* < 0.05). CD4 count is substantially higher among MSM than other population groups (Figure 1a). Specifically, 66% (365/553) MSM has CD4 Cell counts greater than 200, while the proportion in other populations were much lower (FPD: 23% [30/132]; IDU: 31% [361/1164] and heterosexuals: 35% [1919/5,484], Figure 1b). Baseline viral load was 4.73 log10 copies/mL (IQR, 3.69–5.47). A total of 2426 patients were diagnosed as in WHO clinical Stage 1, and 1471, 1775 and 915 at Stage 2, 3 and 4 respectively (Table 1).

### 3.2. Trend of CD4 and Viral Load Level 

Median CD4 level among all ART patients increased from 148 cells/mm^3^ (IQR 41–243) to 344 cells/mm^3^ (IQR 234–477) over the first 42-month of treatment. Out of the 228 patients who received viral load tests, viral load level declined 430 folds from 4.73 log10 copies/mL (IQR 3.69–5.47) at treatment initiation to 2.1 log10 copies/mL (IQR 1.60–3.18) in the same period (Appendix A). About 80.7% (184/228) patients achieved viral suppression within 12 months of treatment.

CD4 level in MSM had a steady increase from 242 cells/mm^3^ (IQR 167–298) to 386 cells/mm^3^ (IQR 292–470) over the first 42-month of treatment. Similarly, heterosexuals and IDU also showed an upward trend from 144 cells/mm^3^ (IQR 40–242) to 354 cells/mm^3^ (IQR 246–485) and 134 cells/mm^3^ (IQR 38–224) to 332 cells/mm^3^ (IQR 213–477) respectively, but the level is substantially less than that of MSM at the study end point. CD4 level in FPD have demonstrated the fluctuation in a narrow range during the follow-up, overall it rose from 86 cells/mm^3^ (IQR 36–181) to 264 cells/mm^3^ (IQR 177–383) (Figure 1a).

### 3.3. Treatment Failures among ART Patients

**Figure 2:**
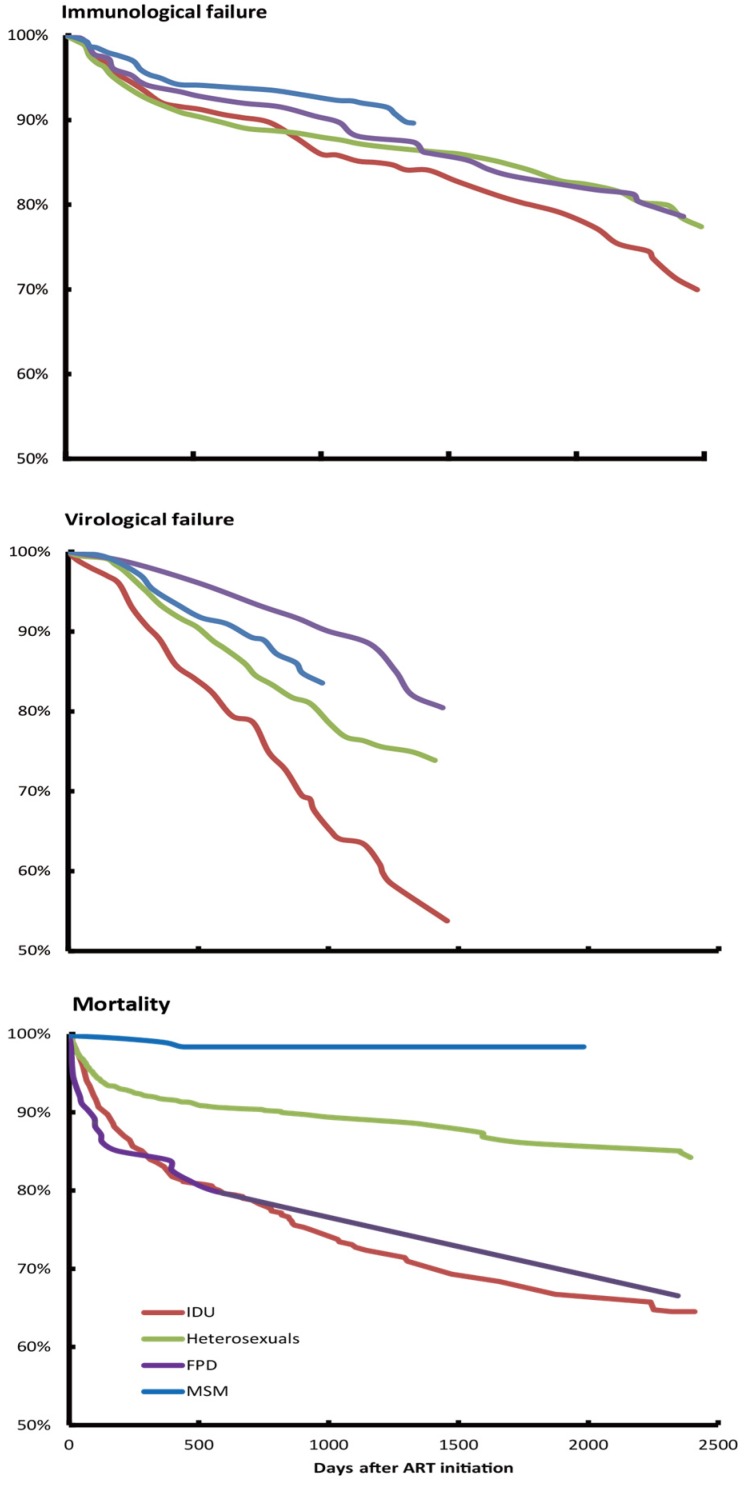
Survival curves for immunological failure, virological failure and HIV-related mortality, stratified by transmission routes.

MSM had consistently the lowest immunological and virological failure rates over time, reached 10.4% and 16.4% at the end of the follow-up. This is followed by FPD (immunological failure: 21.38% [10/47] and virological failure 20.0% [1/5] and heterosexuals (immunological failure: 22.6% [666/2946]; and virological failure: 26.4% [14/53]). Notably, among the 1164 IDU patients, the percentage increased to 30.1% (134/446) among IDU who retained in treatment for at least 42 months. Similarly, virological failure was found among 45.8% (11/24) IDU during the same period (Figure 2).

### 3.4. Mortality during Treatment

A total of 1040 patients (14.2%) deceased during treatment (MSM 12; IDU 329; FDP 29; heterosexual 670). MSM had the highest survival rate (98.3%), followed by the heterosexuals (84.2%) and FPD (66.5%), while IDU had the lowest survival rate (64.5%) at 42 months follow-up (Figure 2).

### 3.5. Significant Factors Associated with Treatment Failure and Mortality

**Figure 3:**
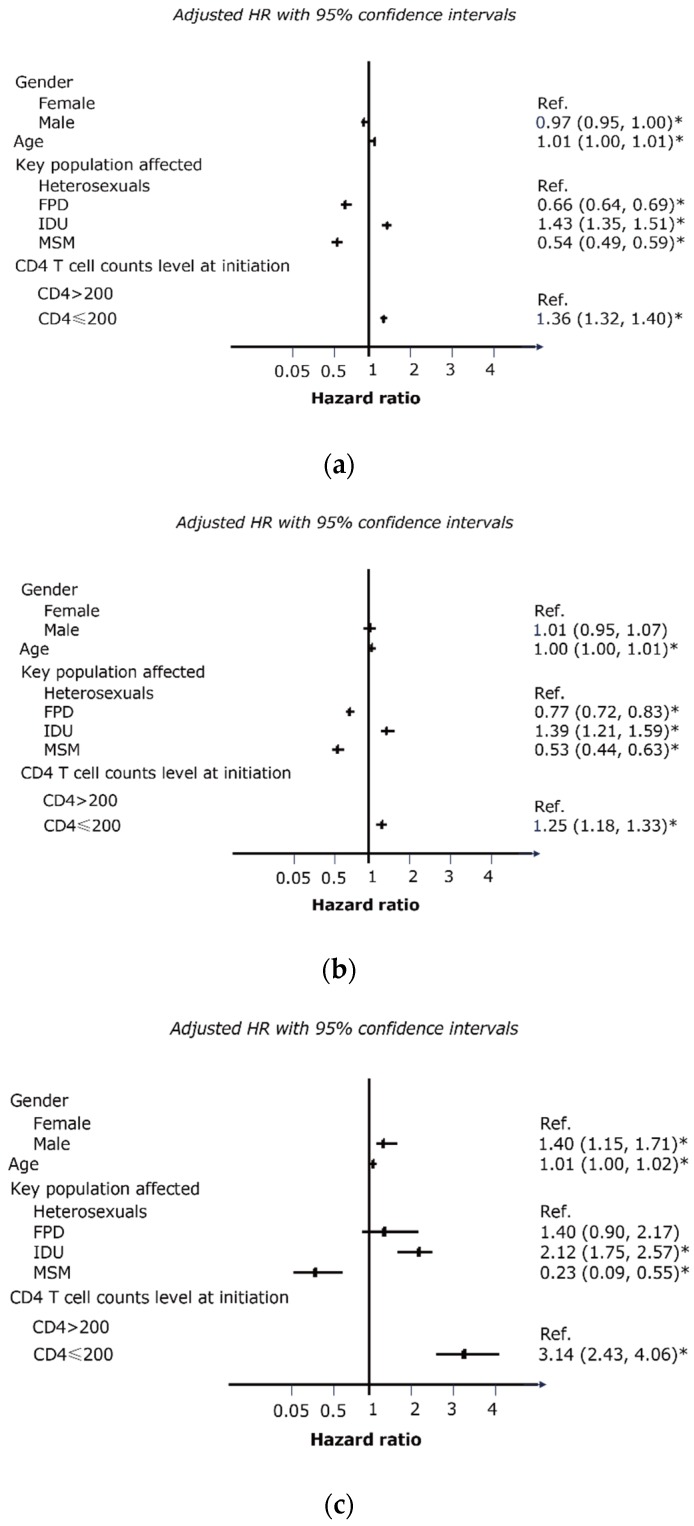
(**a**) Significant hazard factors associated with immunological failure due to HIV infections by cox regression analysis; (**b**) Significant hazard factors associated with virological rate due to HIV infections by cox regression analysis; (**c**) Significant hazard factors associated with HIV-related mortality, by cox regression analysis.

Our adjusted multivariable Cox proportional hazards analysis indicated that being infected through homosexual contacts, plasma donation, and initial ART CD4 counts ≥200 cells/mm^3^ were protective factors against treatment failure. In particular, the rate of immunological failure in IDU, MSM and FPD during the 48-month follow-up was approximately 17%, 8% and 12%, respectively. These corresponded to 1.43 (1.35–1.51), 0.54 (0.49–0.59) and 0.66 (0.64–0.69) times higher risk in comparison with heterosexuals. Similarly, MSM and FPD were 47% and 23% (AHR = 0.53; CI, 0.44–0.63, and AHR = 0.77; CI, 0.72–0.83, respectively) less likely to experience virological failure compared with heterosexuals, whereas IDU was at 39% (AHR = 1.39; CI, 1.21–1.59) higher risk. Patients with CD4 level below 200 cells/mm^3^ had a 35.6% higher risk of immunological failure (AHR = 1.36; CI, 1.32–1.40) and 25% higher risk of virological failure (AHR = 1.25; CI, 1.18–1.33) than otherwise (Figure 3a,b).

Being female, being infected through homosexual contacts, initial ART CD4 counts ≥200 cells/mm^3^ and younger age were protective factors against HIV-related death. Specifically, male patients had a 1.4 times greater risk of death (AHR = 1.40; 95% CI, 1.15–1.71) than female, and the mortality risk in MSM was only about 20% (AHR = 0.23; CI, 0.09–0.55) of the heterosexuals while the risk is more than double in IDUs (AHR = 2.12; CI, 1.75–2.57). Patients with CD4 level below 200 cells/mm^3^ had three times higher risk of death (AHR = 3.14; CI, 2.43–4.06) than otherwise. Aging patients have slightly elevated mortality risk (AHR = 1.01; CI, 1.00–1.02) (Figure 3c).

## 4. Discussion

This retrospective cohort study demonstrated that MSM in Hunan have higher median CD4 T-cell counts at baseline and retained better health outcomes than other populations throughout the course of ART treatment. MSM consistently demonstrated lower rates of immunological failure, virological failure and mortality compared with the patients infected via other routes. The robust CD4 response and viral suppression presented in MSM are comparable to findings of long-term ART patients in developed countries [29,30]. 

Our study confirmed that high CD4 cell level at treatment initiation among MSM can lead to improved treatment outcomes. On the contrary, previous MSM studies in other Chinese settings have consistently reported low testing coverage, which resulted in delayed diagnosis and treatment, and rapid progression to AIDS [19,31,32]. Improved health outcomes among MSM in our study may be due to multiple factors. In particular, the HIV screening program promoted by the Gates and Melinda Foundation, with the cooperative efforts from Hunan CDC under the national “Four Frees and One Care” policy, may have diagnosed a large population of HIV-infected MSM in the province and substantially improved HIV awareness during the study period [24]. Besides, HIV outbreaks in MSM in Hunan are reportedly much later than other coastal provinces in China [33], MSM in Hunan are also better prepared for the epidemic and adhered to ART than other parts of the country [34].

Maintaining high CD4 count at early phrase of treatment plays an important role for subsequent treatment outcomes. This is consistent with previous findings in China [35,36] and internationally [37]. These findings convey a unified message that timely treatment initiation is vitally important for improved treatment outcomes in the long term. The low treatment failure rate in MSM implies a relatively low drug resistance level in this population in Hunan, which concurs with a previous study [22]. In contrast, without targeted interventions, virological failure rates in IDU in Hunan are noticeably higher than that in other parts of China [36,38]. These substantial differences may be attributed to the later ART initiation (only 31% of IDUs initiated on time) and their poor treatment adherence. A separate study reported that 15% IDU patients skipped medication in the last month [35], and that treatment adherence among IDUs is significantly worse than that of non-injecting drug users [39,40]. Psychological distress, strained family relationship, unstable employment and concurrent drug use may all contribute to the inherence [41,42]. Notably, the markedly elevated immunological and virological failure rates in IDU may also relate to their high opportunistic infections rate, because of their high-risk injecting behaviours [43,44,45]. AIDS-defined complications may cause permanent damages to the immune system that cannot be fully recovered even with ART [46,47,48].

The analysis of HIV-related mortality demonstrated similar results. Participants with baseline CD4 <200 cells/mm^3^, mostly IDU, have the lowest survival probability. Similarly, FPD, with lowest median CD4 level at baseline, demonstrated similar survival probability as IDU. As HIV transmission through illegal blood transfusion occurred mostly during the early stage of HIV epidemics in China, FPD likely experienced delayed diagnosis and treatment due to the absence of a national ART program then [49,50]. This may have led to inadequate immune responses to sustain high enough CD4 level to combat against the HIV infection [51,52,53]. In IDUs, additional adverse effects associated with harm from drug addiction may add to the risk of mortality in the HIV+ IDUs. Further, addiction may also lead to sub-optimal treatment adherence in IDUs, thereby counteracting the benefits of ART [54,55]. The antagonism between ART regimens and opioid substitution therapy can also lead to reduced adherence to ART. Male participants are known to be more prone to risk behaviours and less aware of their own health status. In comparison, females are more self-aware and hence more likely to participate in HIV testing and comply with clinical advices to receive timely ART [56,57,58]. This finding concurs with studies conducted in other developing countries [59,60]. In addition, age has a moderate impact on mortality possibly because aging can further weaken the immune system. Although the influence is marginal, given that HIV-infected population is gradually ageing, further studies are still necessary to understand the complications associated with ageing on death in this population.

A number of limitations of this study should be noted. First, the routes of HIV infection were self-reported by the participants; as such, it is difficult to determine the accuracy of the information. In particular, MSM may choose to conceal their sexual identity due to social stigma. Second, most MSM started ART after 2007, so the record of follow-up was not as complete as that for IDU, FPD and heterosexuals. Besides, the record prior to 2007 may be less accurate than more recent data, owing to the immature reporting mechanism then. This may affect data quality of FPD and comparability between study populations. Third, the study design did not capture risk factors for HIV exposure or information on past interventions that participants might have received. This prevents us from investigating the reasons for their early or late diagnoses. Fourth, as a retrospective study, the recorded information is subjected to recall bias by participants. Fifth, only 228 (3.11%) patients received viral testing at baseline. Given the high out-of-pocket cost of the test, this may have led to overestimation of the actual virological failure rate.

## 5. Conclusions

Conclusively, our study indicated that HIV-positive MSM can achieve better treatment outcomes and reduced mortality than other at-risk populations over the long-term course of ART if they have high CD4 count at ART initiation. This conveys an important message, that diagnosis and early treatment are possible in an opaque population that is often stigmatised in a developing country setting.

## Figures and Tables

**Table 1:** Demographic characteristics of Participants.

Characteristics		No. (Percent)
Sex		
	Female	2436 (33.2%)
	Male	4897 (66.8%)
Age, years, median (IQR)		40 (32–49)
Marriage status		
	Single	1489 (20.3%)
	Married	4230 (57.7%)
	Divorced	963 (13.1%)
	Widowed	639 (8.7%)
	Unknown	12 (0.16%)
CD4 at ART Initiation (cells/mm^3^) (IQR)		151 (43–246)
	Female	164 (79–283)
	Male	138 (32–225)
Viral Load at ART Initiation (log10 * copies/mL) (IQR)		4.73 (3.69–5.47)
WHO Stage at ART Initiation		
	Stage 1	2426 (33.1%)
	Stage 2	1471 (20.1%)
	Stage 3	1775 (24.2%)
	Stage 4	915 (12.5%)
	Missing	746 (10.2%)
CD4 group (cells/mm^3^)		
	0–50	1905 (26.0%)
	50–100	870 (11.9%)
	100–200	1699 (23.2%)
	200–350	2372 (32.3%)
	350–500	169 (2.3%)
	>500	48 (0.7%)
	Missing	270 (3.7%)
Days from diagnosis to treatment(IQR)		60 (26–273)
Transmission route		
	Heterosexuals	5484 (74.8%)
	Men who have sex with men	553 (7.5%)
	Injecting drug users	1164 (15.9%)
	Former plasma donor	132 (1.8%)

**Figure A1:**
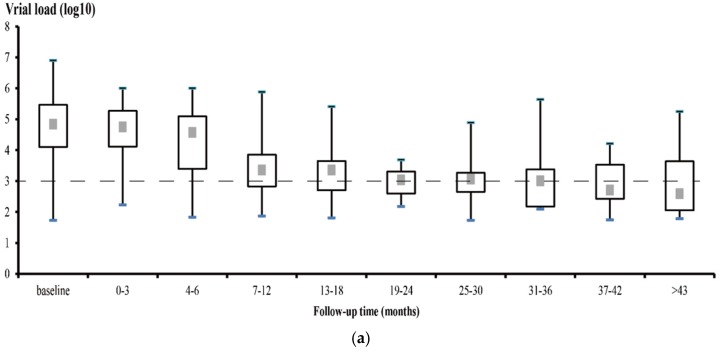

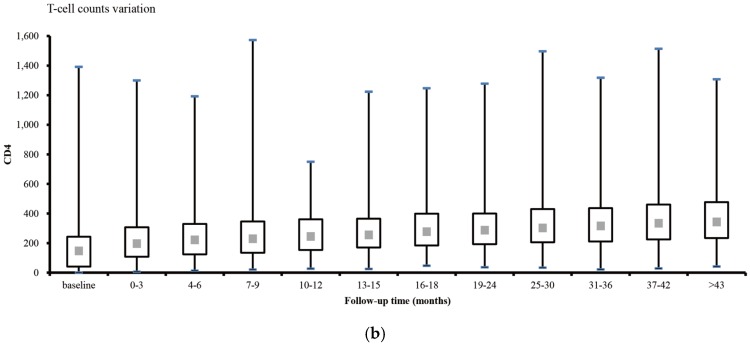
(**a**) Viral load variation during follow-up time after accepting ART; (**b**) CD4 T-cell counts variation during follow-up time after accepting ART. Viral load and CD4 T-cell counts variation during follow-up time after accepting ART.
